# CAR T cell therapy becomes CHIC: “cytokine help intensified CAR” T cells

**DOI:** 10.3389/fimmu.2022.1090959

**Published:** 2023-01-09

**Authors:** Simone Thomas, Hinrich Abken

**Affiliations:** ^1^ Leibniz Institute for Immunotherapy, Div. Genetic Immunotherapy, Regensburg, Germany; ^2^ Department of Internal Medicine III, University Hospital Regensburg, Regensburg, Germany; ^3^ Chair for Genetic Immunotherapy, University Regensburg, Regensburg, Germany

**Keywords:** cytokine, signal-3, T cell activation, chimeric antigen receptor, adoptive cell immunotherapy

## Abstract

Chimeric antigen receptors (CARs) in the canonical “second generation” format provide two signals for inducing T cell effector functions; the primary “signal-1” is provided through the TCR CD3ζ chain and the “signal-2” through a linked costimulatory domain to augment activation. While therapy with second generation CAR T cells can induce remissions of leukemia/lymphoma in a spectacular fashion, CAR T cell persistence is frequently limited which is thought to be due to timely limited activation. Following the “three-signal” dogma for inducing a sustained T cell response, cytokines were supplemented to provide “signal-3” to CAR T cells. Recent progress in the understanding of structural biology and receptor signaling has allowed to engineer cytokines for more selective, fine-tuned stimulation of CAR T cells including an artificial autocrine loop of a transgenic cytokine, a cytokine anchored to the CAR T cell membrane or inserted into the extracellular CAR domain, and a cytokine receptor signaling moiety co-expressed with the CAR or inserted into the CAR endodomain. Here we discuss the recent strategies and options for engineering such “cytokine help intensified CAR” (CHIC) T cells for use in adoptive cell therapy.

## Introduction

Initially most B cell leukemia/lymphoma patients respond favorably to chimeric antigen receptor (CAR) T cell therapy, however, the majority of patients fail to achieve long lasting remission due to the loss of functional CAR T cell capacities and persistence (1). Declining in CAR T cell effector functions represents a major hurdle for the success of CAR T cell therapy in general, bringing up the question whether the signals provided by the current canonical CAR format are sufficient for enduring T cell activation.

According to the concept of stepwise T cell activation, acquisition of effector functions and establishment of long-term T cell memory require the integration of three distinct signals: primary activation (“signal-1”) is transmitted through CD3ζ following T cell receptor (TCR)-mediated antigen recognition; costimulation (“signal-2”) augments “signal-1” through TCR associated signaling receptors like CD28, 4-1BB, or OX40; cytokine support (“signal-3”) as provided by a panel of pro-inflammatory cytokines extends T cell amplification maturation of naïve into effector T cells, and development of a memory T cell compartment (2–4). Not only the quality of each signal but also their chronological sequence upon antigen engagement is crucial for productive T cell activation. Within this concerted action, cytokine help is promoting chromatin remodeling to maintain transcription of numerous genes needed for enhancing or repressing signaling circuits, thereby providing positive and negative signals to the activation pathway. Interleukin (IL) 12, type-1 interferons (IFNs) and γ-cytokines (IL2, IL4, IL7, IL9, IL15, IL21) are predominant third signals for a variety of T cell responses. The individual γ-cytokines mediate different signals through their respective receptors, which is mechanistically due to competing for the common γ chain along with binding to their specific receptor chains and consequently activation of different downstream signaling pathways. Overall, the relative amounts of cytokine receptors and the integration of all cytokine signals that are available during the antigen recognition orchestrates the process of T cell activation and its maintenance.

The currently used 2^nd^ generation CARs deliver “signal-1” and “signal-2”, but not the cytokine based “signal-3” (5). Although activated CAR T cells release a panel of cytokines, including IFN-γ and IL2, their production and cytokine receptor mediated activation declines during continuous antigen engagement below stimulatory levels. In addition, some cytokines, such as IL7, IL12 and IL15, are produced either not or only at low levels by activated T cells, however, are crucial for maintaining CAR T cell effector functions after adoptive transfer. The deficiency creates the need of cytokine supplementation in order to sustain T cell functional capacities and to execute effector functions over an extended period of time.

## Procedures to deliver the cytokine signal to CAR T cells

**Figure 1:**
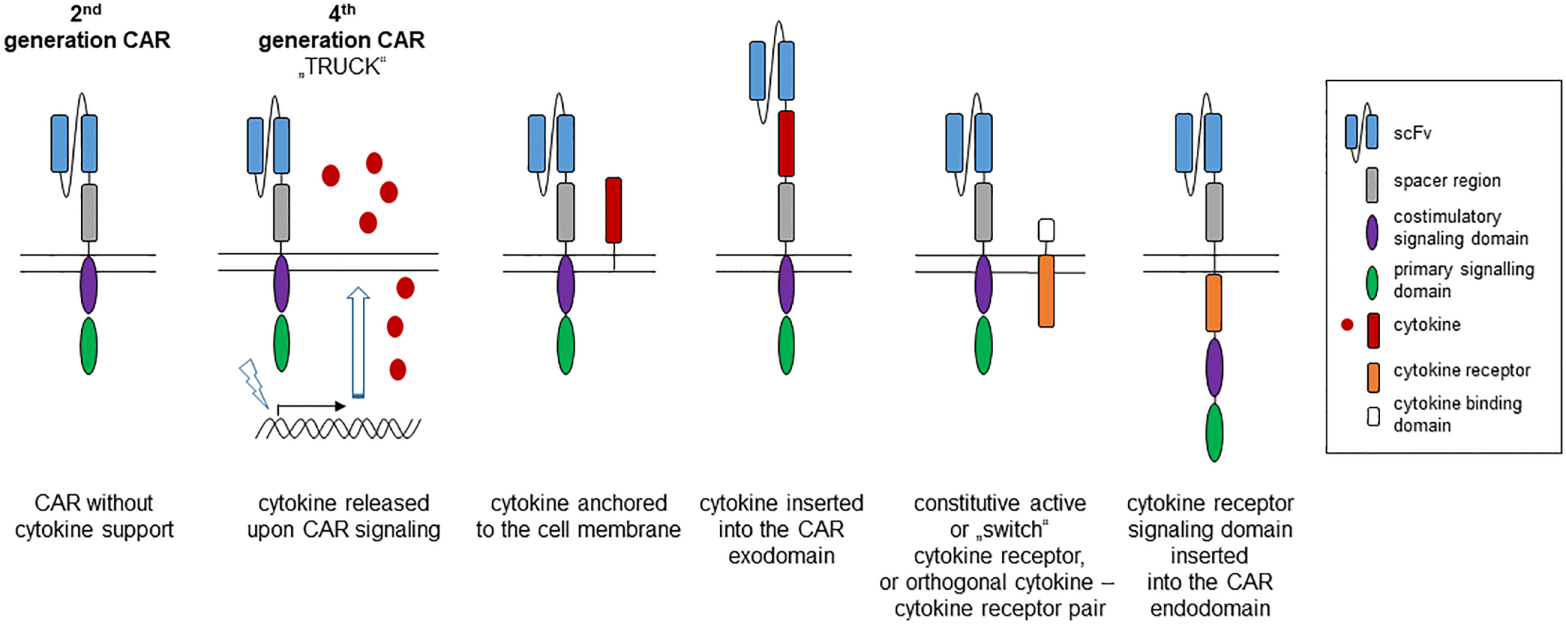
Strategies to provide cytokine help to a “second generation” CAR. The canonic 2^nd^ generation CAR is composed of an antibody derived binding domain, spacer, transmembrane and two linked intracellular signaling domains, one providing the primary activation (“signal-1”) and the other the costimulatory signal (“signal-2”). Support by a transgenic cytokine (“signal-3”) can be made available by CAR triggered synthesis and release of the cytokine (4^th^ generation CAR, so-called TRUCK), by anchoring the cytokine to the cell membrane or by inserting into the CAR exodomain. A cytokine signal can also be delivered by co-expressing a constitutive active cytokine receptor or by inserting the cytokine receptor signaling domain into the intracellular CAR moiety.

Based on the three-signal dogma in T cell activation, cytokines were supplemented during cell therapy to prolong CAR T cell functional capacities. The benefit of cytokine help became exemplarily obvious when supplementing IL7 during CAR T cell treatment strongly supported homeostatic expansion of the CAR T cell compartment (6). Most cytokines are pleiotropic and act on many cell types and impact multiple functional pathways that increases the risk of dose-limiting toxicity when systemically applied. Recent progress in structural biology, molecular engineering and in understanding receptor pharmacology (6), has allowed to develop strategies using the therapeutic potential of cytokine help in the context of adoptive cell therapy in doing so creating “cytokine help intensified CAR” (CHIC) T cells. Technically, several options are currently explored (**Figure 1**):

-cytokine released by engineered CAR T cells (cytokine “TRUCK”),

-cytokine anchored to the CAR T cell membrane,

-cytokine integrated into the CAR extracellular moiety,

-constitutive active cytokine receptor, and

-cytokine receptor signaling domain linked to the CAR intracellular moiety.

Which cytokine and which mode of delivery is efficacious and safe to sustain CAR T cell functional capacities is currently under investigation as outlined below. In particular, IL7, IL12, IL15 and IL18 are explored in various formats on the level of pre-clinical models and in early phase clinical trials. From a more general perspective, signaling through these cytokines is capable to mediate a sustained and balanced activation of different STAT hetero-/homo-dimers or tetramers that interact with the chromatin and impact the expression of multiple transcription factors in a specific fashion resulting in sustained CAR T cell activation.

### Cytokine released by engineered CAR T cells (cytokine TRUCK)

During the last years, strategies were developed to engineer CAR T cells with constitutive or inducible release of cytokines that act in an autocrine or paracrine fashion to orchestrate immune cell function in a specific fashion. The concept of CAR inducible expression of a transgenic cytokine is also known as TRUCK (“T cells redirected for universal cytokine-mediated killing”) or “4th generation CAR” (7, 8); CARs with constitutive or inducible cytokine release are summarized under the term “armored CARs” (9).

Among the currently explored cytokines, IL12 has the capacity to take a key role in boosting CAR T cell therapy due to its capacity to strongly activate cytolytic T and NK cells and to orchestrate the Th1 response. Taking advantage to these properties, we and other groups engineered IL12 TRUCK cells to deliver IL12 to the CAR T cells in an autocrine fashion and to the tumor infiltrating immune cells in a paracrine fashion (10, 11). The capacity of CAR T cell released IL12 to muster a host innate anti-tumor response became obvious when antigen-negative cancer cells, invisible to CAR T cells, were eradicated in a mixed tumor by infiltrating macrophages, that respond to IL12 (10), and by endogenous T cells through increased antigen processing and presentation (11). Several groups currently translate the concept of CAR T cells with constitutive IL12 release into clinical trials (e.g. NCT02498912; NCT03932565) (**Table 1**). However, preliminary evaluation revealed a high incidence of hemophagocytic lymphohistiocytosis macrophage activation-like syndrome (12) that forced the development of inducible cytokine release triggered by CAR signaling (10, 13). Along this line, EGFR-specific CAR T cells with inducible IL12 are currently explored in a trial for the therapy of metastatic colorectal cancer (NCT03542799).

**Table 1:** Selected CAR T cell trials exploring the safety and efficacy of added cytokine or cytokine receptor (“signal-3”).

Cytokine, cytokine receptor	CAR target	Disease	clinicaltrials.gov identifier
IL7 + CCL19 IL7 + CCL19 IL7 + CCL19 IL7 + CCL19 IL7 + CCL19 or IL12 IL12 IL12 IL15 IL15 memIL15 IL15, IL21 IL15 IL15 IL15R agonist (NKTR-255) IL18 IL4Rα/IL2Rβ C7R C7R IL8R	CD19 CD19 GPC3 BCMA Nectin-4/FAP EGFR MUC16ecto GD2 GD2 CD19 GPC3 GPC3 GPC3 CD19 CD19 ErbB2 GD2 GD2 CD70	B cell lymphoma Diffuse large B cell lymphoma Hepatocellular carcinoma Multiple myeloma Solid tumors Colorectal cancer Ovarian cancer Neuroblastoma, osteosarcoma Lung cancer B cell leukemia/lymphoma Pediatric solid tumors Hepatocellular carcinoma Pediatric solid tumors B cell malignancies B cell lymphoma/chronic lymphatic leukemia Head & Neck squamous cell carcinoma Brain tumors Solid tumors Glioblastoma	NCT03929107 NCT04381741 NCT03198546 NCT03778346 NCT03932565 NCT03542799 NCT02498912 NCT03721068 NCT05620342 NCT03579888 NCT04715191 NCT05103631 NCT04377932 NCT03233854 NCT04684563 NCT01818323 NCT04099797 NCT03635632 NCT05353530

memIL15, membrane anchored IL15; C7R, constitutive active IL7 receptor.

As examples for γ-cytokines, T cells with a GD2-specific CAR were engineered to release IL7 (14) or IL15 (15, 16), the latter CAR T cells are currently explored for the treatment of neuroblastoma (NCT03721068, NCT03294954). CD19-specific CAR T cells engineered to release IL7 showed improved killing and expansion in pre-clinical models, however, less persistence compared to IL15 engineered CAR T cells (17). IL7 co-expressed with C-C motif chemokine ligand-19 (CCL19) or CCL21 produced an improved T cell memory response along with increased chemotaxis to the tumor lesion and enhanced amplification (18–20). Early clinical trials are currently evaluating the safety and efficacy of IL-7/CCL19 engineered CAR T cells against lymphoma (NCT03929107, NCT04381741), multiple myeloma (NCT03778346), and solid tumors (NCT03932565). One drawback of the strategy is that the IL7 receptor (IL7R) is downregulated upon repetitive IL7 stimulation thereby limiting the efficacy of the autocrine stimulatory circuit. The deficit is aimed to be overcome by constitutive expression of the IL7R (21, 22) or of a composite constitutive active cytokine receptor (23) that acts independently of the cytokine.

There is a strong rationale to use IL18 and IL36, both belonging to the IL1-superfamily of cytokines, in the framework of TRUCK cells since both, like IL12, can not only activate engineered CAR T cells but also host immune cells to recognize tumors. Transgenic IL18 induced in an autocrine fashion a T-bet^high^ FoxO1^low^ signature in CAR T cells that are characterized by an improved cytolytic response with augmented amplification and cytokine release in pre-clinical models (24, 25). In a paracrine fashion, IL18 cooperates with the Th1 response, augmenting the innate response by attracting NK cells, and inducing a productive host T cell response by antigen spreading and reducing the number of repressor cells (25, 26). IL18 releasing CAR T cells are currently explored for the treatment of CLL and non-Hodgkin’s lymphoma in a trial at the University of Pennsylvania (NCT04684563). In a German academic, multi-center phase I trial, TRUCK cells releasing inducible IL18 upon GD2 engagement are shortly explored for the therapy to pediatric and adult GD2^+^ tumors. Like IL18, co-expressed IL36γ acts in an autocrine fashion to augment expansion and persistence of CAR T cells (19), and also acts in a paracrine fashion promoting the maturation of antigen presenting cells and supporting tumor recognition by host T cells through antigen spreading.

### Cytokine anchored to the CAR T cell membrane

IL15 has the capacity to improve CAR T cell persistence and to promote the stem cell memory T cell (T_SCM_) development (27), providing the rationale to anchor IL15 directly to the T cell membrane. This is the more supported since the autocrine stimulatory effect of membrane anchored IL15 seems to be stronger than that of secreted IL15 (28). IL15 predominantly acts by trans-presentation through its receptor; specifically, IL15 was fused to the IL15Rα by a flexible linker mimicking trans-presentation of IL-15 in context with its receptor (29). Trans-presented IL15/IL15Rα provided an enduring signal to CD8^+^ T cells without much triggering other endogenous immune cells (30) and sustained maintenance of T_SCM_ CAR T cells in a pre-clinical model to a greater extent than treatment with soluble IL15/IL15Rα (29). The example demonstrated that restricting the cytokine signal to the CAR T cell itself minimizes systemic toxicities.

### Cytokine integrated into the extracellular CAR moiety

An alternative strategy to restrict the cytokine activity to the CAR T cell is based on inserting the cytokine into the extracellular CAR moiety while preserving both the CAR and cytokine function. Using IL12 as an example, we inserted a functionally active, single-chain p40-p35 IL12 variant into the extracellular CAR moiety (31). The specific CAR architecture allows functional activities of both domains, the cytokine and the tumor targeting scFv; redirected T cell activation remains strictly antigen-dependent while activation of STAT4, being an IL12 downstream event, is improved. More strikingly, the IL12-CAR conveys NK cell-like capacities to engineered T cells, along with a down-regulated Th2 response, making them capable to mediate antigen-independent killing. Other cytokines, in particular γ-cytokines, may likely be active in reprogramming CAR T cell functions when inserted into the CAR exodomain.

### Constitutive active or triggered cytokine receptor

A constitutive active cytokine receptor can mimic the presence of a specific cytokine by delivering the downstream activation signal independently of binding of the respective cytokine. In a specific application, constitutive active IL7R, as created by forced homo-dimerization of the IL7R α-chains, triggers STAT5 and phospho-inositol-3-kinase (PI3K) signaling and improves anti-tumor activity of CAR T cells in the absence of IL7 (23). The result is similar to that of covalently linked IL7 to the respective IL7R (32). While these are prototypes, it demonstrates the power of a modified cytokine receptor to provide a constitutive active signal to the engineered T cell.

In modification of the strategy, a composite receptor was engineered that binds a specific cytokine and delivers through a heterologous downstream domain a signal that is physiologically not provided by the respective cytokine receptor. A so-called “switch receptor” converts binding of a repressive cytokine into an activating signal and vice versa. As downstream signaling moiety, common γ-chain receptors like IL2R, IL15R, IL7R are mostly used that signal primarily through STAT5, activate PI3K and, except for IL7R, activate the MAP kinase pathway. A number of such cytokine switch receptors are explored in the meantime including an IL4/IL7 receptor that provides IL7 signaling upon IL4 binding (33, 34). A TGFβ/IL7 switch receptor converts the repressive TGFβ signal into a stimulatory IL7 signal by upregulating pATK and Bcl-xL (35); a GM-CSF binding receptor provides IL18R signaling (36). We previously reported a CAR triggered artificial autocrine loop by the CAR induced release of IL7 that binds to an artificial IL7 binding receptor linked to the intracellular IL2R β-chain that stimulates the MAP kinase pathway resulting in improved, autocrine CAR T cell amplification (22). Such inducible cytokine releasing T cells with co-expression of the corresponding, synthetic receptor create an artificial autocrine circuit as long as the CAR engages its cognate antigen; loss of antigen and finally CAR triggering switches off the autocrine loop and thereby the “signal-3” for sustained activation.

To avoid serious adverse event of systemically applied of IL-2 and to avoid activation of IL-2 receptor (IL-2R) expressing bystander cells like regulatory T (Treg) cells, an orthogonal (ortho) murine IL2 and IL2Rβ pair was engineered through a directed evolution strategy (37); a human orthogonal IL2 – IL2Rβ pair was recently reported (38). Ortho-IL2 selective binds to the ortho-IL2Rβ receptor and produces downstream IL2 receptor signaling in engineered T cells without substantial signaling through the natural IL2 receptor. CAR T cells engineered to co-express the ortho-IL2Rβ expanded specifically upon stimulation through ortho-IL2 in a pre-clinical model improving anti-tumor capacities. CAR T cells administered in a suboptimal dose were rescued by ortho-IL2 to execute a curative response instead of an otherwise failed anti-leukemia response. The example demonstrates that applying a selective artificial cytokine – cytokine receptor pair allows to amplify CAR T cell engraftment and functional capacities in a tunable and target-specific fashion.

### Cytokine receptor signaling chains integrated into the CAR intracellular moiety

Most approaches so far relied on co-expressing membrane-anchored cytokines or cytokine receptors. Instead of integrating the cytokine into the extracellular CAR domain, the signaling moiety of a cytokine receptor was linked to the CAR intracellular signaling moiety. As an example, a CAR with combined intracellular CD28-IL2Rβ chain triggered superior amplification and effector functions of the engineered T cells (39). In continuation of the concept, JAK/STAT binding domains were incorporated into the CAR intracellular domain (39, 40). A broader application of the strategy, however, is likely limited by the fact that the different signaling domains compete in their required proximities to the cell membrane, and thereby in their position within the CAR endodomain, in order to sufficiently recruit their proprietary downstream mediators.

## Discussion

Pre-clinical studies support the benefit of transgenic cytokines like IL15 and IL7 in augmenting the CAR T cell anti-tumor response; first clinical trials are evaluating safety and efficacy of the approach (**Table 1**). Engineering the CAR T cell with the respective cytokine is aimed at avoiding systemic side effects as well as providing a constant cytokine trigger as long as the CAR T cell persists. A co-expressed membrane-anchored cytokine, a CAR with integrated cytokine or a constitutive active cytokine receptor on the CAR T cell surface would ideally fulfill this need. Loss of CAR and thereby cytokine expression would result in diminished and finally loss of redirected functional capacities and T cell persistence.

On the other hand, there is a more general concern that constitutive γ-cytokine stimulation may result in uncontrolled CAR T cell activation and amplification (41) and, consequently, toxic levels of TNFα and IFNγ may moreover increase the risk for cytokine release syndrome (CRS). While IFN-γ is required for successful tumor destruction (42), supra-physiological IFN-γ levels may accumulate particularly in case of co-expressed IL18 that can cooperate with IL12 or IL15 to further increase IFN-γ production. Basically, CRS can nowadays be recognized at an early stage and clinically managed; the actual CRS risk upon application of cytokine engineered CAR T cells is not sufficiently known. There are currently no data available based on a clinical head-to-head comparison of supplemented vs secreted vs membrane bound cytokines in CAR T cell therapy with respect to sustaining CAR T cell activities while avoiding systemic immune activation.

While triggering CAR T cell activation through cytokines, the degree in CAR T cell amplification and the release of pro-inflammatory cytokines needs to be fine-tuned and balanced to avoid systemic inflammation; this basically applies to all cytokines that play a crucial role in pro-inflammatory reactions and can potentially contribute to augment severity of adverse events. Addition of a suicide switch, e.g., by adding inducible caspase-9, may be required as it allows rapid and sufficient depletion of the IL15 CAR T cells in a pre-clinical model (15) and is added to CAR T cells with IL15 release in a clinical trial (NCT03721068).

Cytokine help of CAR T cells can be used to not only strengthen and prolong CAR T cell activation, but also to locally orchestrate the adoptive and host immune cell response towards tumors in a more sophisticated fashion. Physiologically, the inflammatory immune response goes through a phase of initiation, immune cell recruitment and amplification and finally cessation of inflammation. These stages are ideally mirrored by a stepwise activation of CAR T cells in the targeted tissue and by recruiting and activating host immune cells to sustain the anti-tumor response. In this context, CAR T cells with “signal-3” are not only understood as booster for CAR T cells but also as instructors of the immune environment for sustaining the anti-tumor response. For successful tumor elimination, it will be crucial how efficient cytokine activated CAR T cells can recruit and activate the endogenous, pre-existing anti-tumor response towards a more balanced and lasting fashion. The concept may be developed further by implementing sequential expression of a defined set of cytokines during different stages of the CAR triggered immune response.

## Data availability statement

The original contributions presented in the study are included in the article/supplementary material. Further inquiries can be directed to the corresponding author.

## Author contributions

All authors listed have made a substantial, direct, and intellectual contribution to the work and approved it for publication.
